# Contribution of the Roman rat lines/strains to personality neuroscience: neurobehavioral modeling of internalizing/externalizing psychopathologies

**DOI:** 10.1017/pen.2023.7

**Published:** 2023-10-04

**Authors:** Alberto Fernández-Teruel, Toni Cañete, Daniel Sampedro-Viana, Ignasi Oliveras, Rafael Torrubia, Adolf Tobeña

**Affiliations:** Medical Psychology Unit, Department of Psychiatry & Forensic Medicine, Faculty of Medicine & Institute of Neurosciences, Autonomous University of Barcelona, 08193 Bellaterra, Barcelona, Spain

**Keywords:** externalizing disorders, internalizing disorders, neurobiology, reward sensitivity, Roman high- and low-avoidance rats, threat sensitivity

## Abstract

The Roman high-avoidance (RHA) and low-avoidance (RLA) rat lines/strains were established in Rome through bidirectional selection of Wistar rats for rapid (RHA) or extremely poor (RLA) acquisition of a two-way active avoidance task. Relative to RHAs, RLA rats exhibit enhanced threat sensitivity, anxiety, fear and vulnerability to stress, a passive coping style and increased sensitivity to frustration. Thus, RLA rats’ phenotypic profile falls well within the “internalizing” behavior spectrum. Compared with RLAs and other rat strains/stocks, RHAs present increased impulsivity and reward sensitivity, deficits in social behavior and attentional/cognitive processes, novelty-induced hyper-locomotion and vulnerability to psychostimulant sensitization and drug addiction. Thus, RHA rats’ phenotypes are consistent with a “disinhibiting externalizing” profile. Many neurobiological/molecular traits differentiate both rat lines/strains. For example, relative to RLA rats, RHAs exhibit decreased function of the prefrontal cortex (PFC), hippocampus and amygdala, increased functional tone of the mesolimbic dopamine system, a deficit of central metabotropic glutamate-2 (mGlu2) receptors, increased density of serotonin 5-HT2A receptors in the PFC, impairment of GABAergic transmission in the PFC, alterations of several synaptic markers and increased density of pyramidal immature dendrític spines in the PFC. These characteristics suggest an immature brain of RHA rats and are reminiscent of schizophrenia features like hypofrontality and disruption of the excitation/inhibition cortical balance. We review evidence supporting RLA rats as a valid model of anxiety/fear, stress and frustration vulnerability, whereas RHA rats represent a promising translational model of neurodevelopmental alterations related to impulsivity, schizophrenia-relevant features and comorbidity with drug addiction vulnerability.

## Once upon a time, some rats left Rome – the many roads leading to it took them everywhere

**Figure 1.**
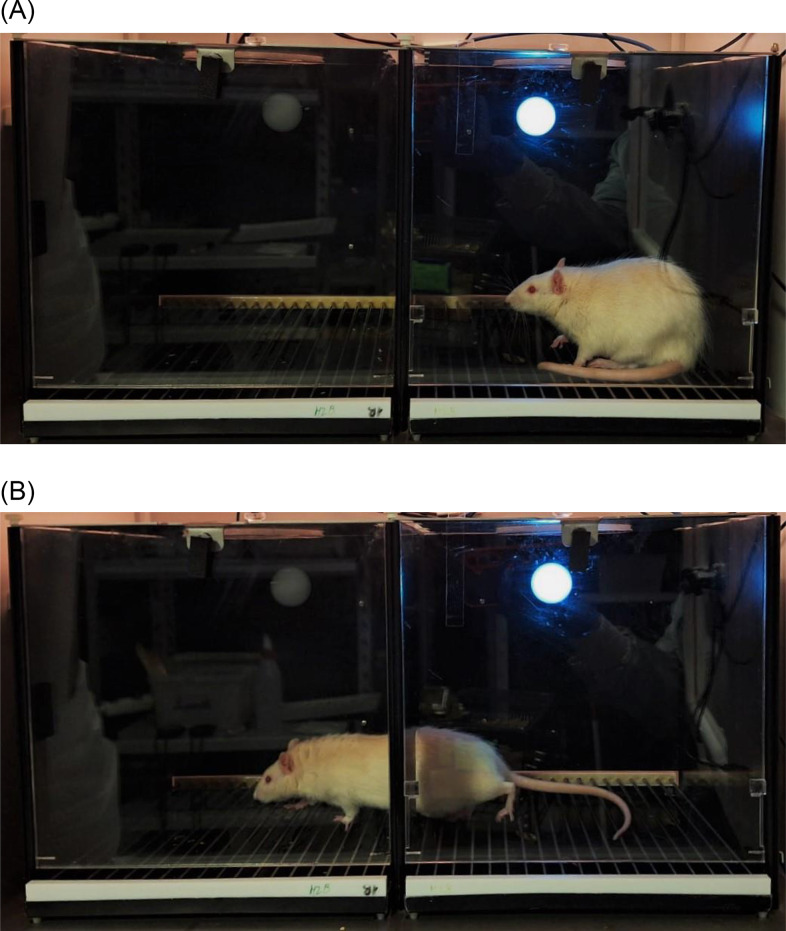
Two-way active avoidance acquisition in the shuttle box (Medical Psychology Unit, Dept. Psychiatry & Forensic Medicine, Autonomous University of Barcelona). The rat is crossing from the right compartment to the opposite one to escape/avoid the shock but is also faced with the memory of having to escape from the left compartment on the previous trial. For further explanation see text.

The Roman high-avoidance (RHA) and low-avoidance (RLA) rat lines were established in Rome through bidirectional selection and outbreeding of Wistar rats for rapid (RHA) or extremely poor (RLA) acquisition of a two-way active avoidance task (TWAA; Figure 1) (Bignami, 1965). The two lines were then moved to Birmingham, UK (e.g., Broadhurst & Bignami, 1965). Two inbred strains were established in London (in 1974) and, derived from them, two inbred Roman sub-strains were established in Nijmegen (Durcan, Wraight & Fulker, 1984). Outbred sublines, from the Birmingham stock, were established in Canada (e.g., Satinder, 1971), France (e.g., Delacour, Houcine & Monmaur, 1978) and in Zurich, Switzerland, in 1972 (the RHA/Verh and RLA/Verh sublines; e.g., Driscoll & Bättig, 1982). Outbred sublines from the Zürich stock were established in Cagliari, Italy (e.g., Giorgi et al., 1994), Bordeaux, France (e.g., Castanon & Mormède, 1994), Geneva, Switzerland (Steimer & Driscoll, 2003) and in Germany (e.g., Schwegler et al., 1997). Inbred RHA and RLA strains, generated through brother/sister mating of the respective Swiss/Verh sublines, were established in Barcelona in 1995 (e.g., Driscoll et al., 1998). Over 270 international articles have been published on the Roman rats (or using them as part of broader studies) during the past fifty-five years. They have been used for psychological, neurobiological and genetic research purposes in over 20 different laboratories around the world (see Supplementary Table S1, in Fernández-Teruel et al., 2021), and continue to be very productive, as shown by the over 35 papers published in the past five years.

In the TWAA task rodents can learn to escape/avoid an aversive stimulus (unconditioned stimulus, US; usually a foot shock) that is preceded by a warning signal (conditioned stimulus, CS; usually a tone, a light, or both) by shuttling between opposite compartments (Figure 1). After a few initial CS–US pairings, the TWAA generates approach-avoidance conflict, with the appearance of context- and CS-conditioned freezing (e.g., Fernández-Teruel & Tobeña, 2020). This tendency to freeze (i.e., passive avoidance: Tendency to stay freezing in the present compartment, where they receive the CS) predominates in the initial trials, as the subject learns that the opposite compartment is also threatening (i.e., approach-avoidance conflict), because in that compartment he has also received a foot shock. As training and CS–US associations progress, the subject learns that the US can be overcome by shuttling to the opposite compartment, first by escaping (when the shock US is present) and later on by avoiding it (i.e., by crossing to the opposite compartment when the CS is still on and the US has still not been presented).

The RLA rat line/strain presents high levels of freezing to the CS and the context (during the inter-trial intervals), and very low rate of active avoidance responses and inter-trial crossings. The RHA line/strain displays very low levels of freezing, very rapid active avoidance acquisition and high levels of inter-trial activity (Broadhurst & Bignami, 1965; Satinder, 1971; Driscoll & Bättig, 1982; Fernández-Teruel et al., 1991; Escorihuela et al., 1995; reviewed by Fernández-Teruel et al., 2021; Giorgi et al., 2019).

## Selection for poor two-way active avoidance task acquisition led to behaviorally inhibited, high “neuroticism” and “internalizing” Roman low-avoidance rats

Thus, when facing different types of threatening situations, RLA rats are predominantly high freezers, displaying behavioral inhibition and a passive coping style (opposite to the trait profiles of RHA rats, as we will see below; see also reviews by Driscoll et al., 1998; Fernández-Teruel et al., 2021; Steimer & Driscoll, 2003; Giorgi et al., 2019). These trait differences generalize to many between-strain phenotype differences in conditioned and unconditioned tests/tasks reflecting coping style of fear-, anxiety- and depression-related responses. For example, relative to RHA rats, RLAs present increased signs of anxiety in typical anxiety tests such as the Vogel’s punished-drinking, the elevated zero- and plus-maze and light-dark box tests among others; they also show enhanced frustration-related (Amsel, 1992) responding under reward devaluation, and heightened stress-linked endocrine and depressive-like responses (reviewed by Fernández-Teruel et al., 2021; Giorgi et al., 2019). Remarkably, RLAs also exhibit increased context- and cue-conditioned freezing and, like high trait-anxiety humans (Andión et al., 2013), RLAs display enhanced stress- and fear(cue)-potentiated startle (Aguilar, Gil, Tobeña, Escorihuela & Fernández-Teruel, 2000; López-Aumatell et al., 2009). From the perspective of the Research Domain Criteria (RDoC) framework (for Review, see Michelini, Palumbo, DeYoung, Latzman & Kotov, 2021) such a profile suggests that RLA rats are mainly driven by “negative valence” (e.g., Michelini et al., 2021) (see section A in Table 1). In fact, they have enhanced sensitivity to threat/distress and behavioral inhibition, which is compatible with a high “neuroticism” and low “extraversion” personality profile (see section C in Table 1) (e.g., Gray & McNaughton, 2000). In sum, RLAs’ behavioral profile seems to be dominated (and driven) by negative affectivity, which is a core characteristic of human internalizing disorders (e.g., depression, anxiety- and stress-related disorders; see e.g., Michelini et al., 2021). Thus, RLA rats phenotypes seem to fall within the domain of “Internalizing” spectra and “Fear” and “Distress” sub-factors in terms of the “Hierarchical Taxonomy of Psychopathology” (HiTOP; Michelini et al., 2021) system (see section B in Table 1).

**Table 1.** Overview of neurobehavioral traits of RLA and RHA rats, correspondence with RDoC domains, the HiTOP psychopathology spectra (Michelini et al., 2021) and the “Big Five” and “Eysenck-Gray” personality factors

Line/Strain	(A) RDoC system (Domains)	(B) HiTOP system (Spectra, sub-factors & Symptom components)	(C) Personality (“Big Five” traits & “Eysenck-Gray”- related factors)	(D) Main Neuroscience-based traits (RDoC domains)
**RLA**	**Negative valence:** (+); Fear, Anxiety, Sustained threat (depressive-like) effects, Frustrative non-reward **Cognitive systems:** (+); Attention, Declarative memory, Working memory **Arousal Systems:** (+); Threat-induced (anxious) Arousal	“**Internalizing”** Sub-factors: **FEAR, DISTRESS** ***Symptoms/phenotypes:*** (+) Sensitivity to punishment (anxiety); Behavioral inhibition (+) Stress-induced helpless (depression-like) behavior; (+) Threat-induced freezing, Passive avoidance; (+) Enhanced stress- and fear-potentiated startle (+) Enhanced reward-devaluation-induced frustration	**(+) Neuroticism** -High **“Sensitivity to punishment”**/ high BIS activity (*REFS:* *Gray & McNaughton 2000*; Giorgi et al., 2019) **(-) Extraversion**	(+) function of the hippocampus (HPC), amygdala (AMY) and prefrontal cortex (PFC), (+) CRF expression in PVN of the hypothalamus, BNST and AMY. (+) AMY-dependent HPA-axis (ACTH, corticosterone) and prolactin responses to stress. (-) 5-HT function in FC (-) BZR function in cortex *(REFS:* Giorgi et al., 1994, 2003, 2019; Bentareha et al., 1998; Carrasco et al., 2008; Meyza et al., 2009; Tapias-Espinosa et al., 2019, 2023)
**RHA**	**Positive valence:** (+); Reward Responsiveness, Reward Valuation **Cognitive systems:** (-) Attention, Working memory, Declarative memory **(-) Systems for Social Processes:** Detachment **Sensorimotor Systems (altered)**	“**Disinhibited Externalizing”:** Sub-factors: **SUBSTANCE ABUSE** ***Symptoms/phenotypes:*** (+) Impulsivity; Behavioral disinhibition; Hyperactivity; Vulnerability to drug addiction (+) Delay discounting; Risky decision-making in a rat gambling task (+) Schizophrenia spectrum symptoms (-) Social interaction preference (-) Prepulse inhibition & Latent inhibition (-) performance in spatial and non-spatial working memory tasks, spatial reference learning/memory and cognitive flexibility (+) Sensitivity to reward (+) Habit (compulsive) learning	**(+) Extraversion** -High **“Sensitivity to reward”**/high BAS activity (*REFS:* *Gray & McNaughton 2000*; Giorgi et al., 2019) -High **“Impulsive-Sensation/Novelty seeking”** (*REFS:* Fernández-Teruel et al., 1992; Moreno et al., 2010; Giorgi et al., 2019; Bellés et al., 2021a-b, 2023; Dimiziani et al., 2021; Zuckerman et al., 1974; Siegel & Driscoll *1996*; Sampedro-Viana et al., 2021; Oliveras et al., 2023)	(+) mesolimbic DA activity, and altered striatal dopamine activity. (-) D2/3 receptors in Str (+) D1 receptors in PFC and Str (+) Homer1 & Neuregulin1 expression in PFC. Transcriptome analysis in the FC shows deficient maturation. (-) PFC, HPC and AMY function and volume (+) 5-HT2A in PFC (-) mGlu2R in PFC, HPC, Str Augmenting pattern of cortical VEPs (*REFS:* Fernández-Teruel et al., 2021; Siegel et al., 1993; Guitart-Masip et al., 2006; Meyza et al., 2009; Tournier et al., 2013; Klein et al., 2014; Fomsgaard et al., 2018; Elfving et al., 2019; Tapias-Espinosa et al., 2019, 2023; Bellés et al., 2021a; Sánchez-González et al., 2021; Sønderstrup et al., 2023 *)*

(+), (-); increased/improved, decreased/impaired, respectively. For reasons of space, the references are placed in columns C and D, but they also apply to columns A and B. See text for abbreviations.

### Neuroanatomical and neuro-functional profiles of Roman low-avoidance rats

At neurochemical and molecular levels, selection of outbred rats for extremely divergent rates of acquisition of TWAA, or comparison of RLA vs RHA rats, has been shown to be associated with differential expression of many genes related to neural and synaptic function at the hippocampal and amygdala levels (Díaz-Morán et al., 2013a, 2013b; Sabariego et al., 2013). In this regard, and consistent with descriptions of the neuropsychology of anxiety (Gray & McNaughton, 2000; McNaughton & Corr, 2004), relative to RHA rats the RLAs display a more pronounced neural activation of the hippocampus (HPC) and amygdala (AMY), as reflected by increased c-Fos expression when exposed to different novelty-based approach-avoidance conflict situations (Meyza, Boguszewski, Nikolaev & Zagrodzka, 2009). In addition, the activity of the phospholipase C signaling cascade is higher in the HPC of RLA vs. RHA rats (Sallés et al., 2001). Accordingly, the volume of the HPC and AMY is larger (Río-Álamos et al., 2019), and the neuronal density is higher in both brain areas of RLA vs. RHA rats (Gómez et al., 2009; Garcia-Falgueras, Castillo-Ruiz, Put, Tobeña & Fernández-Teruel, 2012).

Authors from our group have studied the relationship between functioning of Gray and McNaughton’s “Behavioural Inhibition System” (BIS; as measured by the “Sensitivity to Punishment” scale; Torrubia, Ávila, Moltó & Caseras, 2001) and HPC and AMY volumes in adult healthy human subjects. Consistent with the theory (Gray & McNaughton, 2000), and with the above findings from RLA (vs. RHA) rats, the authors found that BIS scores were highly correlated with HPC and AMY gray matter volume, thus suggesting that behavioral inhibition (resulting from increased activity of the BIS) is associated with enhanced HPC and AMY function (Barrós-Loscertales et al., 2006, and references therein).

Importantly also, the activation of the medial PFC (mPFC; as measured by c-Fos expression) following exposure to various novelty tests or to a prepulse inhibition (PPI) session, as well as mPFC volume, is also enhanced in RLA rats relative to their RHA counterparts (Meyza et al., 2009; Río-Álamos et al., 2019; Tapias-Espinosa et al., 2019, 2023) (see section D in Table 1). On the other hand, alterations of the GABA-A/benzodiazepine receptor and levels of endogenous benzodiazepine-like molecules have also been found in cortex and HPC of RLA (vs. RHA) rats (Giorgi et al., 1994; Driscoll et al., 1995; Bentareha et al., 1998) (see section D in Table 1).

In sum, the enhanced activity of HPC, mPFC and AMY, together with more stress-responsive hypothalamus (HPA-axis) and bed nucleus of the stria terminalis (Steimer & Driscoll, 2003; Carrasco et al., 2008; Río-Álamos et al., 2017; see section D in Table 1), seem to be consistent with theory (Gray & McNaughton, 2000) and with the behaviorally-inhibited and high threat-sensitivity profile of RLA rats (Giorgi et al., 2019) (sections A-B in Table 1).

## Selection for rapid two-way active avoidance task acquisition led to behaviorally disinhibited, reward sensitive and “externalizing” Roman high-avoidance rats

Compared with RLA and other laboratory strains/lines, the RHAs are behaviorally disinhibited, exhibiting a consistent low-anxious profile in unconditioned and conditioned tests of anxiety or fear (Fernández-Teruel et al., 2021; Steimer & Driscoll 2003; Giorgi et al., 2019). They are hyperactive and highly exploratory, novelty/sensation seeking, impulsive and present impairments of attention, sensorimotor gating (PPI), latent inhibition, startle habituation, working memory and cognitive flexibility (see sections A-B in Table 1). Remarkably, RHA rats exhibit increased locomotor and nucleus accumbens (NAcc) dopaminergic (DA) sensitization to chronic psychostimulants and other abused drugs (morphine, ethanol), enhanced cocaine self-administration and reinstatement after extinction, enhanced preference for ethanol and natural rewards and increased functional tone of the mesolimbic DA system (see sections B and D in Table 1). Vulnerability to drug addiction and schizophrenia are thought to share some core features and/or mechanisms. In particular, for instance, the increased functional tone of the mesolimbic DA system is considered to be at the core of both schizophrenia and drug addiction and is present in RHA rats relative to RLAs (Giorgi et al., 2019; Fernández-Teruel et al., 2021).

Collectively, the above profiles are consistent with RHA rats being driven by “positive valence” processes (see section A in Table 1) and characterized by high reward sensitivity (and impulsivity) and impairments in the cognitive domain, which are compatible with a personality dominated by “Impulsivity/Sensation seeking” traits (Zuckerman, Murtaugh & Siegel, 1974; Siegel, Sisson & Driscoll, 1993; Gray & McNaughton, 2000; Giorgi et al., 2019) (see section C in Table 1). Impulsivity, or impairment of behavioral control (i.e., behavioral inhibition deficit), is often a cause of both maladaptive behaviors directed toward an individual’s environment and interference in life functioning. These traits are typically associated with attention/cognition impairments, impulsive choices/actions (i.e., premature responses or actions without foresight, often leading to adverse or maladaptive consequences) and hyperactivity. If combined with high reward/sensation seeking, they relate to risky behavior and vulnerability to substance use and abuse (e.g., review by Giorgi et al., 2019, and references therein). All these aspects converge in RHA’s behavioral profile (Fernández-Teruel et al., 2021; Giorgi et al., 2019). Hence, the RHA rats seem to fall within the “Disinhibited Externalizing” spectra and “Substance Abuse” sub-factor, and likely also within the “Thought disorder” spectra (Michelini et al., 2021; see section B in Table 1), as they present various schizophrenia-linked traits, such as, e.g., hyperactivity, relative asociality, and impairments of inhibitory control (impulsivity) and attentional/cognitive processes (Fernández-Teruel et al., 2021; Giorgi et al., 2019) (see Table 1).

### Neurobiological and molecular profiles of Roman high-avoidance rats

Recent studies have shown that RHA rats present alterations of pre-/postsynaptic markers and trophic factors in the PFC and/or HPC (e.g., neuregulin1, homer1, synaptophysin and brain-derived neurotrophic factor/BDNF) that have been linked with glutamatergic dysfunction, PFC maturation and schizophrenia (Elfving et al., 2019; reviewed by Fernandez-Teruel et al., 2021). In addition, RHA rats exhibit increased DA D1 receptors in the PFC and the NAcc, augmented NMDA2B receptors in the PFC, enhanced 5-HT2A receptors in PFC and HPC, a dramatic deficit of mGlu2 receptors in PFC, HPC and striatum (Elfving et al., 2019), and increased density of immature dendritic spines in the PFC (Sánchez-González et al., 2021). This is overall consistent with the evidence of lowered function and volume of the PFC and HPC in RHA rats (e.g., Meyza et al., 2009; Río-Álamos et al., 2019; Tapias-Espinosa et al., 2019, 2023). These molecular/synaptic markers and neuro-functional profiles strongly suggest that RHA rats have immature (i.e., adolescent-like) PFC and (likely) HPC, which lead to impaired top-down inhibitory control (e.g., of the mesolimbic DA system) (Sønderstrup et al., 2023). Collectively, these profiles cohere with their behavioral/cognitive alterations, and with their drug-seeking and schizophrenia-linked trait profiles (Fernandez-Teruel et al., 2021; Giorgi et al., 2019).

It has been known since the late 1990s that the reward-sensitive and impulsive RHA rats are characterized by an enhanced functional tone of the mesolimbic DA system, as shown, for instance, by *in vivo* DA microdialisis in the NAcc following sensitization to psychostimulants (e.g., Giorgi et al., 2007; Tournier et al., 2013; Bellés et al., 2021a; reviewed by Giorgi et al., 2019; and Fernández-Teruel et al., 2021). Remarkably, these early findings are consistent with more recent findings obtained in healthy humans. Thus, reward sensitivity, as measured by the “Behavioural Activation System” (BAS) trait scale (Carver & White, 1994; Gray & McNaughton, 2000), is positively correlated with activity (measured through fMRI) of the dorsomedial and ventral striatum (equivalent to the NAcc in rats) during a gambling task involving reward-punishment conflict (Costumero et al., 2016). Hence, it is noteworthy that the findings on DA function in RHA (vs. RLA) rats translate to humans.

## Epigenetic and “genotype x environment” interaction effects on personality/psychopathology

Certain traumas, or stressful or positive events, particularly those experienced at young ages, can lead to markers (e.g., DNA methylation) being placed on genes, changing their functional expression. These are the subject of study of epigenetics (e.g., Francis, Diorio, Liu & Meaney, 1999; Bahari-Javan et al., 2017). Among other things, epigenetics deals with the study of “why”, “when” and “how” the markers that environmental life events place on genes can lead to traceable changes in the development of the nervous system, biological and mental processes and behavior.

Neonatal handling (NH; a neonatal stimulation treatment administered to rats or mice during the first weeks of life) and environmental enrichment (EE; administered for several months from puberty to adulthood) induce stable long-lasting changes in the personality profile and HPA-axis stress responses in RLA as well as many other laboratory rats, so that treated rats become enduringly much less threat- and stress-sensitive (e.g., see Fernández-Teruel, Escorihuela, Castellano, González & Tobeña, 1997; Fernández-Teruel et al., 2002a, 2002b; Río-Álamos et al., 2017, 2019, and references therein) than their non-handled counterparts. NH treatment of offspring induces improved dams’ maternal behavior, which in turn induces epigenetic changes in the HPC involving the nerve growth factor-inducible protein A (NGFI-A) transcription factor and glucocorticoid receptors, and thus HPA-axis function (Francis et al., 1999; Weaver et al., 2007). Importantly, neonatal handling leads to an enduring reduction of HPC and AMY volume in RLA rats, which correlates with the NH-decreased threat sensitivity and anxious/fear behavior (Río-Álamos et al., 2017, 2019).

Many “genotype x environment” interactions have been reported for the effects of NH and/or EE treatments on RHA/RLA rat behavior and neuroendocrine traits (for reviews, see Fernández-Teruel et al., 2002a, 2021; and Río-Álamos et al., 2017, 2019, and references therein). NH and EE treatments also influence many aspects of personality of RHA rats. For example, NH improves attentional (PPI) and cognitive functions in this strain, and NH and/or EE long-lastingly improve spatial cognitive processes of RHA rats and enhance novelty- and/or substance-seeking behavior in both Roman rat strains (e.g., Fernández-Teruel et al., 1997, 2002b; Río-Álamos et al., 2019; Bellés, Dimiziani, Herrmann & Ginovart, 2021b; Bellés, Arrondeau, Urueña-Méndez & Ginovart, 2023).

Regarding adverse early environmental events, we have found that social isolation rearing (from pre-puberty to adulthood) affects RHA/RLA rat behavior in a manner dependent upon the strain (i.e., “genotype x environment” interactions). Thus, in RHA (but not RLA) rats this isolation enhances anxiety and hyperactivity; and impairs PPI and PFC-/HPC-related reference memory, thus further increasing the schizophrenia-related impairments of RHA rats (Oliveras et al., 2016; Sánchez-González et al., 2020).

Still, in the context of adverse early events (i.e., early life stress), caregiving maltreatment produces epigenetic changes in the rat adolescent brain (e.g., HPC, AMY), which could have relevance to adolescent mental health and behavior (Doherty et al., 2016). In relation to psychopathology, Bahari-Javan et al., (2017) showed that (i) early life stress (ELS)-induced schizophrenia-like behavioral and synaptic phenotypes in mice were associated with increased expression of histone-deacetylase-1 (*Hdac1*; one of the epigenetic processes) in PFC, possibly linked to DNA methylation changes (another epigenetic process) and (ii) administration of an HDAC1 inhibitor reversed these schizophrenia-like phenotypes. Importantly, *Hdac1* blood levels were also increased specifically in patients with schizophrenia who had been exposed to ELS as compared with patients with schizophrenia who didn’t suffer ELS (Bahari-Javan et al., 2017). Thus, by using a translatable animal model of induction of schizophrenia-relevant behavioral features, it was suggested that a biological (epigenetic) factor might be a relevant antecedent of at least some types of (environmentally-linked) schizophrenia, to the point that “…analysis of *Hdac1* expression in blood could be used for patient stratification and individualized therapy” (Bahari-Javan et al., 2017, p. E4686).

The above examples illustrate that both personality and associated neurobiological and psychopathological processes can be enduringly influenced by environmental factors in a manner dependent upon genetic background (“genotype x environment” interactions). Epigenetics tells us that these environmental influences on proneness to particular types of pathological symptom clusters will operate in many cases through environmentally-induced changes in the epigenome. Thus, manipulating the environment in animal models, at different neurodevelopmental stages and in both (negative and positive) directions (Fernández-Teruel, 2021), and exploring neurobiological and epigenetic changes and their relation with variation of personality and psychopathology-linked phenotypes, might be crucial for a coherent/integrative and translatable development of personality neuroscience and neuroscience-based psychopathology.

## What more can the Roman rats tell us about personality neuroscience and psychopathology?

Unlike selection of many other rat lines/strains for simpler innate behavioral traits, selection of the Roman rats for their (high/low) ability to acquire the functionally complex two-way avoidance task involves a variety of processes. Two-way avoidance involves (at least) CS and context Pavlovian threat conditioning (related to passive avoidance and freezing – i.e., passive coping- tendencies), instrumental escape conditioning, one-way active avoidance conditioning, locomotor activity in a threat-conditioned context (e.g., inter-trial crossings) and flight responses (undirected active responses searching alternative escape routes).

It might be argued that the markedly enhanced capacity of RHA rats to acquire TWAA learning might be due to the fact that they make better associations between the fear-eliciting CS and the aversive US (Mowrer & Lamoreaux, 1946). Studies of Pavlovian threat conditioning (i.e., classically-conditioned freezing) and fear-potentiated startle seem to argue against that contention, since in these paradigms RHAs exhibit both lowered conditioned freezing and decreased potentiated startle responses (respectively) relative to RLA rats (López-Aumatell et al., 2009; see further references in Fernández-Teruel et al., 2021). Importantly, also, the ability to acquire one-way active avoidance responding does not differ in RHA vs. RLA rats when safety time is relatively long (30 s), i.e., when approach-avoidance conflict is low. Conversely, when safety time is drastically reduced (1 s), and thus approach-avoidance conflict is high, RHA rats acquire one-way active avoidance faster than RLA rats (Torres et al., 2007). These findings collectively suggest that fear-eliciting CS–US associations are stronger in RLA than RHA rats, thus prompting the former strain to develop enhanced Pavlovian reactions (i.e., freezing, passive avoidance), which interfere with active (escape/avoidance) responses, particularly when intensity of approach-avoidance conflict (and thus, threat) is high (Fernández-Teruel & McNaughton, 2023).

In sum, the various processes involved in TWAA acquisition, as mentioned above, together (or in interaction) with parental strain (Wistar) and individual genetic characteristics and/or vulnerabilities (e.g., threat sensitivity vs. reward sensitivity), have led to two rat lines/strains that differ in a large number of stable neurobehavioural traits. These are not specific for two-way active avoidance, nor are only related to defensive responses in the face of threat (threat sensitivity) or approach-avoidance conflict, but extend to changes in responses to positive reinforcers (reward sensitivity, drug addiction), impulsivity and attentional/cognitive processes (Fernández-Teruel et al., 2021; Giorgi et al., 2019).

Hence, the threat-sensitive RLA rats present comorbidity with fear and distress, within the “Internalizing” spectra (see Table 1; Michelini et al., 2021). The extensive neurobiological and behavioral characterization carried out with RLA rats (e.g., Fernández-Teruel et al., 2021; Steimer & Driscoll, 2003; Giorgi et al., 2019) makes them a very promising tool for personality neuroscience and psychopathology research. They might be particularly useful, for example, to study the neurobiological underpinnings of behavioral disinhibition (and top-down inhibitory control) mechanisms (e.g., PFC, HPC), relations among top-down inhibitory control and (good) cognitive performance, anxiety, stress-induced depression, anxious depression, reward-devaluation-induced frustration, environmental influences on these symptoms/phenotypes and epigenetic changes underlying these influences.

The high reward-sensitive and behaviorally-disinhibited RHA rats present comorbidity of symptoms associated with “Thought disorder” (i.e., schizophrenia-linked phenotypes) and “Disinhibited externalizing” (substance abuse, impulsivity) spectra of the HiTOP system (Michelini et al., 2021; Table 1). Their behavioral/attentional/cognitive profile, combined with the extensively characterized neurobehavioural and molecular schizophrenia- and drug-addiction-linked phenotypes make them a unique tool to investigate comorbidity of schizophrenia-relevant features, drug abuse (and addiction) and PFC-, HPC- and NAcc-linked cognitive/behavioral alterations. As suggested by recent gene expression findings (e.g., Elfving et al., 2019; Sánchez-González et al., 2021; Sønderstrup et al., 2023), the RHA rats can be a useful tool for understanding the neural maturational trajectory leading to the behavioral and cognitive disturbances associated with schizophrenia and perhaps comorbid traits/symptoms, such as drug-seeking/abuse and impulsivity. In addition, this would allow us to investigate whether early “therapeutic” interventions (e.g., infantile and/or juvenile enriched stimulation, such as NH and EE treatments, combined or not with ELS) might modify these neuro-molecular pathways (or factors) and phenotype expression.

In summary, some examples of the heuristic and translational potential of the Roman rat models are the following:

1. In the RHA and RLA strains, HPC and AMY volume positively correlate with behavioral inhibition in anxiety-related tests (Río-Álamos et al., 2017), consistent with what has been reported between a BIS measure (Torrubia et al., 2001) and volume of these two regions in humans (Barros-Loscertales et al., 2006).
2. Also, in line with human studies showing a positive association between a BAS-trait measure and activity of the striatum during a gambling task (Costumero et al., 2016), behavioral disinhibition (impulsivity) and novelty seeking in Roman rats are predicted by striatal function (measured by amphetamine-induced DA release; Tournier et al., 2013; Bellés et al., 2021a; see Giorgi et al., 2019).
3. The anxious (and alcohol-non-preferring) RLA rats are more sensitive to reward loss-induced frustration than the low-anxious (and alcohol-preferring) RHA rats. Reward loss (appetitive extinction) enhances ethanol preference in RLA (but not RHA) rats, similar to enhanced alcohol consumption (i.e., emotion-induced “self-medication”) in humans experiencing sudden anxiogenic/stressful conditions. Pre-training in a partial reinforcement (PR) instrumental program, a treatment that leads to development of resilience to (reward) loss-induced anxiety, suppresses loss-induced ethanol preference in RLAs in parallel to increasing their frustration tolerance (Cuenya et al., 2012; Manzo et al., 2015). This makes RLA (vs. RHA) rats a translatable model to study innate differences in, and mechanisms of “frustration tolerance”, vulnerability to negative emotion-induced self-medication (and substance use/abuse) and neurobehavioral mechanisms mediating resilience to loss-induced anxiety/frustration.
4. Excessive anxiety of RLA rats in different tests/tasks is attenuated by anxiolytic drugs and increased by known anxiogenic drugs (see Supplementary Table S2 in Fernández-Teruel et al., 2021).
5. Augmenting of cortical visual-evoked potentials (VEPs) is considered a specific neurophysiological marker of high sensation-novelty seeking (and disinhibited) personality in humans. Remarkably, similar to what has been observed in disinhibited sensation-novelty seeker humans and cats, the RHA rats are cortical VEP augmenters, whereas RLA and Wistar rats are VEP-reducers like non-disinhibited humans (Zuckerman et al., 1974; Siegel et al., 1993; Siegel & Driscoll, 1996).
6. Novelty seeking in RHA/RLA rats is inversely associated with postsynaptic dopamine D2 receptor availability in striatum, and similar findings have been reported in humans (reviewed by Giorgi et al., 2019). Consistently, in a large sample of healthy and distressed humans the activity of the ventrolateral PFC and ventral striatum, during an ‘‘uncertain reward expectancy’’ paradigm, was positively related to impulsive-sensation seeking. This is also in line with the enhanced functional tone of the mesolimbic dopaminergic pathway (including the NAcc) in the impulsive-sensation/novelty seeker RHA rats (see Giorgi et al., 2019).
7. In RHA (but not RLA) rats there is a high positive correlation between cortical 5-HT2A receptor density and premature (impulsive) responses in the “five choice serial reaction time” task. This is consistent with findings from studies in rodents and humans in which the pharmacological blockade of the 5-HT2A receptor produces reductions of several types of impulsive responses, and with the notion that 5-HT2A receptors may facilitate some forms of impulsive behavior (reviewed by Klein et al., 2014, and Giorgi et al., 2019).
8. Similar to untreated patients with schizophrenia, RHA rats exhibit enhanced cortical 5-HT2A receptor density and a dramatic deficit of mGlu2 receptors, which has been proposed to be a crucial factor for the neurodevelopmental alterations and the rich schizophrenia-related phenotypes of this rat strain (Fernández-Teruel et al., 2021).
9. Both schizophrenia symptoms and drug addiction are related to increased mesolimbic dopaminergic function in humans and in animal models (e.g., reviewed by Giorgi et al., 2019). Consistently, relative to the non-drug preferring RLA rats, RHAs exhibit schizophrenia-related phenotypes and vulnerability to drug addiction, which are paralleled by enhanced functional tone of the mesolimbic dopaminergic system in RHAs (reviewed by Giorgi et al., 2007, 2019; Dimiziani et al., 2019).

A considerable number of pharmacological studies, with anxiolytic, antidepressant and anxiogenic drugs, or with psychostimulants, pro-psychotic and antipsychotic compounds, among others, have been carried out in the Roman rat lines/strains (e.g., Oliveras et al., 2017; Sampedro-Viana et al., 2023; see reviews by Fernández-Teruel et al., 2021, and Giorgi et al, 2019, and further references). While these studies globally give support to the predictive validity of RLAs as a model of anxiety (i.e., threat sensitivity) and RHAs as a model of schizophrenia-relevant phenotypes and drug-addiction vulnerability (i.e., reward sensitivity), much more psycho- and neuro-pharmacological research is still necessary. Future efforts should focus on (i) expanding the number of relevant behavioral phenotypes affected by drugs in both rat strains, (ii) exploring neurobiological/molecular mechanisms affected by treatments shown to be effective in changing particular phenotypes and, (iii) extending behavioral, pharmacological and neurobiological research to both sexes (Fernández-Teruel et al., 2021).

Collectively, the findings reviewed here provide an overview of the major traits that distinguish RLA vs. RHA rats, from internalizing to externalizing spectra, from neuroticism- and threat-sensitivity-linked vulnerabilities (RLA) to impulsivity and reward sensitivity-related traits (RHA), from good attention/cognitive capacities (RLA) to schizophrenia-linked phenotypes (RHA). Our aim has been to highlight (also giving several examples of translatability) that these two rat strains/lines may constitute important tools for translational research on personality neuroscience and neuroscience-based psychopathology.
